# Adolescent and young adult research across the HIV prevention and care continua: an international programme analysis and targeted review

**DOI:** 10.1002/jia2.26065

**Published:** 2023-03-23

**Authors:** Samantha Calabrese, Matt Perkins, Sonia Lee, Susannah Allison, Gina Brown, Patrick Jean‐Philippe, Nahida Chakhtoura, Jack Moye, Bill G. Kapogiannis

**Affiliations:** ^1^ Maternal and Pediatric Infectious Disease Branch Eunice Kennedy Shriver National Institute of Child Health and Human Development, National Institutes of Health Bethesda Maryland USA; ^2^ Office of Portfolio Analysis, Division of Program Coordination, Planning, and Strategic Initiatives, Office of the Director National Institutes of Health Bethesda Maryland USA; ^3^ Developmental and Clinical Neuroscience of HIV Prevention and Treatment Branch National Institute of Mental Health National Institutes of Health Bethesda Maryland USA; ^4^ Office of AIDS Research National Institutes of Health Bethesda Maryland USA; ^5^ Medical Affairs, HIV Prevention Gilead Sciences, Inc. New York City New York USA; ^6^ Division of AIDS National Institute of Allergy and Infectious Diseases National Institutes of Health Bethesda Maryland USA

**Keywords:** adolescents, adolescent health, global health, healthcare delivery, HIV, review

## Abstract

**Introduction:**

Human immunodeficiency virus (HIV) continues to rise in young people among low‐ and middle‐income countries (LMIC). The US National Institutes of Health (NIH) supports the largest public investment in HIV research globally. Despite advancements in the last decade, adolescents and young adults (AYA) remain underrepresented in research to improve HIV prevention and care. We undertook a programme analysis of NIH grants and conducted a targeted review of linked publications on international AYA research across the HIV prevention and care continuum (HPCC) to inform new initiatives to address the needs of AYA in these settings.

**Methods:**

NIH‐funded grants from 2012 to 2017, pertaining to AYA in LMIC, and evaluating areas of HIV prevention, care and/or treatment were identified. A systematic review of publications limited to funded grants was performed in two waves: 2012–2017 and 2018–2021. The review included a landscape assessment and an evaluation of NIH‐defined clinical trials, respectively. Data on outcomes across the HPCC were abstracted and analysed.

**Results:**

Among grant applications, 14% were funded and linked to 103 publications for the analytic database, 76 and 27 from the first and second waves, respectively. Fifteen (15%) wave 1 and 27 (26%) wave 2 publications included an NIH‐defined clinical trial. Among these, 36 (86%) did not target a key population (men who have sex with men, drug users and sex workers) and 37 (88%) were exclusively focused on sub‐Saharan Africa. Thirty (71%) publications addressed at least one HPCC milestone. Specific focus was on milestones in HIV prevention, care or both, for 12 (29%), 13 (31%) and five (12%) of publications, respectively. However, few addressed access to and retention in HIV care (4 [14%]) and none included microbicides or treatment as prevention. More focus is needed in crucial early steps of the HIV care continuum and on biomedical HIV prevention interventions.

**Discussion and Conclusions:**

Research gaps remain in this portfolio across the AYA HPCC. To address these, NIH launched an initiative entitled Prevention and Treatment through a Comprehensive Care Continuum for HIV‐affected Adolescents in Resource Constrained Settings (PATC^3^H) to generate needed scientific innovation for effective public health interventions for AYA affected by HIV in LMIC.

## 1 INTRODUCTION

As a leader in biomedical and public health research, the US National Institutes of Health (NIH) supports the largest public investment in HIV research globally. Advances in the field of human immunodeficiency virus (HIV) have transformed HIV into a manageable chronic condition. However, adolescents and young adults (AYA) ages 10–24 years remain disproportionately affected by HIV [1, 2]. In 2017, young people accounted for 15% of all people with HIV and 37% of new HIV infections [1]. Among resource‐constrained settings, this population continues to face immense challenges.

Today, an estimated two million adolescents globally are currently with HIV, with 85% in sub‐Saharan Africa (SSA) [3, 4]. Contributions to HIV prevalence among AYA stem from perinatally and sexually acquired HIV [5, 6]. This is because increased antiretroviral therapy (ART) availability has improved the survival of children with perinatal HIV infection (PHIV) into adolescence [7] and new infections continue to be sexually acquired by susceptible AYA.

AYA with HIV have some of the worst HIV outcomes [8], including uncontrolled viremia, which increases the risk for onward transmission of their HIV [9 – 11]. The expansion of ART in this population requires a differentiated approach tailored to the diverse needs and unique issues of AYA [11]. Many AYA with PHIV in low‐and‐middle‐income countries (LMIC) who may not have benefited from in‐country perinatal HIV prevention programmes with intensive infant follow‐up and testing enter care after being diagnosed during routine clinic visits, hospital admissions or as part of research [12]. As late presenters, AYA with PHIV are often at higher risk of morbidity and mortality, severe immunosuppression and may have growth or pubertal delays, and opportunistic infections [13 – 15]. Regardless of perinatal or sexual HIV acquisition, such additional problems may compound the challenges AYA face during a developmentally vulnerable time characterized by an evolving autonomy, poor impulse control and risk‐taking, peer pressure and substance use, which can lead to care non‐adherence and ART failure [15, 16].

AYA with HIV often bear the responsibilities associated with their care, including transitions from paediatric to adult services, which further adversely impacts their health outcomes [17, 18]. Tailored health services for this age group can reduce overall risks and improve engagement and retention in care [19, 20]. As AYA diagnosed with HIV transition to and engage in adult care services, diagnostic and care structures must work synergistically to adapt to their health needs [17, 21]. AYA with HIV face specific challenges, including managing ones’ illness, disclosure to others and self, stigma, and familial and economic stressors [22].

Recent HIV advances include accomplishments in prevention among individuals at risk and care for those with HIV. However, these successes have been highly influenced by behavioural and structural determinants of health, such as non‐adherence, substance use, food insecurity, justice system involvement and access to AYA‐focused health services [23 – 27]. These factors can be systematically addressed using the HIV prevention and care continuum (HPCC) as a care model. The HPCC consists of successive stages or milestones at which individuals with or at risk of HIV engage in prevention, care and treatment services and is used as an outcomes‐oriented model for targeting HIV‐related care and engagement interventions for HIV‐uninfected individuals to remain HIV negative and for individuals with HIV to achieve and maintain viral suppression [28 – 30].

Although expanding access to HIV prevention, care and treatment services has resulted in significant progress towards ending the HIV epidemic, inadequate access to comprehensive sexual and reproductive health services as part of the HPCC remains a barrier to mitigating the growing epidemic in AYA [31]. Limited resources in LMIC can lead to challenges in the implementation of evidence‐based interventions that make it difficult to address unique risks confronted by AYA. To address this gap, the NIH‐supported Adolescent HIV Prevention and Treatment Implementation Science Alliance (AHISA) was initiated to improve implementation in low‐resource settings [32]. Preliminary input from these efforts spurred a programme analysis of grants supported from 2012 to 2017 and an internal targeted review of their linked publications to further inform the trans‐NIH research priorities on the care continuum for AYA. From this, the Prevention and Treatment through a Comprehensive Care Continuum for HIV‐affected Adolescents in Resource Constrained Settings (PATC^3^H) consortium was justified and launched in 2018 to address the needs of AYA in these settings (Additional File S1) [33, 34]. Presented here are analyses of the grants awarded from 2012 to 2017 and their linked publications from 2012 to 2021.

## 2 METHODS

### 2.1 Search methods

#### 2.1.1 Phase I: Grant application data and review

**Figure 1:**
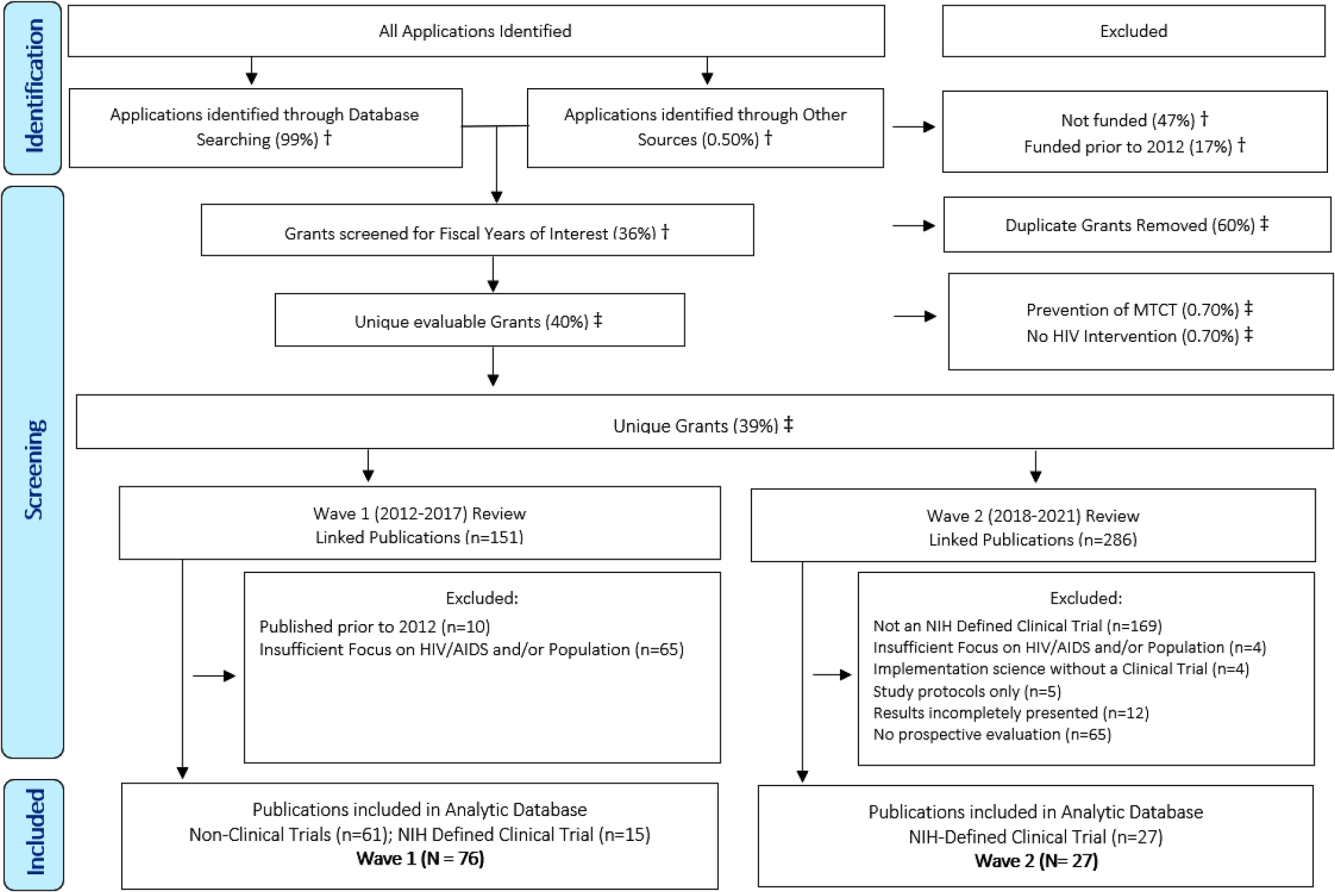
PRISMA flow diagram for the selection of US National Institutes of Health grant applications and their linked publications about the adolescent and young adult (AYA) HIV prevention and care continuum (HPCC) in low‐to‐middle income settings. Abbreviation: MTCT, mother‐to‐child transmission. † Denominator: all applications, ‡ Denominator: grants screened.

Grant applications were identified through searching an internal NIH‐supported database (Figure 1). Applications were included if: (1) the planned project start dates were from 2012 through 2017; (2) the major focus of work was on AYA 10–24 years, inclusive, as the population of interest; (3) the research was planned in international (resource‐limited) settings; and (4) the proposed projects focused on HIV prevention, care and/or treatment. Grants supporting large HIV clinical trial networks and/or prevention of mother‐to‐child transmission (PMTCT) of HIV were excluded.

Among all applications that met the selection criteria, 99% were identified using the NIH database and 0.50% by NIH programme staffs’ review of their respective portfolios. Among these applications, 47% were excluded because they were not funded; an additional 17% received funding prior to 2012 and were also excluded. The remaining 36% with funding initiated or continued during 2012–17 were screened to validate relevance and uniqueness. Of those, 60% were duplicates representing annual non‐competing renewal application records and were removed, and 1.4% were additionally excluded due to insufficient focus on HIV and/or population of interest (e.g. PMTCT in infants) leaving 39% of grants funded during 2012–17 that were unique and relevant to this targeted review.

To explore the contributions to knowledge disseminated by each grant, linkages to their cited publications were ascertained using the Scientific Publication Information Retrieval and Evaluation System (SPIRES), which contains PubMed publication records reporting on research funded by NIH and is updated daily [35].

#### 2.1.2 Phase II: Publication and data review

The contributions of each grant to knowledge disseminated were evaluated across two waves: Wave 1 publications were from 2012 to 2017 and included a broad research landscape analysis of various study designs (e.g. case control, qualitative, systematic review, clinical trial, etc.), whereas evaluations of wave 2 papers were from 2018 to 2021 and focused only on NIH‐defined clinical trials [36].

##### 2.1.2.1 Wave 1: Research landscape assessment

Publications were pulled on 2 January 2018 and reviewed individually by eight subject matter experts to ensure there was a substantial focus on the AYA population, and that geography and HIV outcomes of interest were addressed. Reviewer compendiums were created based on the total number of linked publications. Publications that had an insufficient focus on HIV or AYA were excluded from the final analytic database.

Of the 151 linked publications, 10 were published prior to 2012 and were excluded. A total of 141 publications from 2012 to 2017, inclusive, were evaluated by the reviewers, of which 65 had an insufficient focus on HIV/AIDS and/or inappropriate population focus and thus excluded. The analytic database of publications included 76 manuscripts (54%) for detailed evaluation in wave 1, including 15 manuscripts on NIH‐defined clinical trials.

##### 2.1.2.2 Wave 2: NIH‐defined clinical trial evaluation

Given the paucity of wave 1 publications reporting on NIH‐defined clinical trials targeting AYA HPCC outcomes, wave 2 focused specifically on papers reporting outcomes of primary, secondary and exploratory aims from original and any ancillary analyses of clinical trials reporting on prospective evaluations. Papers on multi‐arm clinical trials were included only if results were presented by arm. Publications were pulled on 14 February 2022, and a similar approach to wave 1 was followed. Reviewer compendiums were created based on the total number of linked publications. Publications were screened and reviewed by two subject matter experts to ensure population focus, HIV outcomes and geography warranted inclusion. A total of 286 linked publications from 2018 to 2021, inclusive, resulted from the unique grant portfolio.

Of the linked 286 publications, 259 papers were excluded for reporting results that did not include an NIH‐defined clinical trial, not reporting any results (e.g. study protocol papers), not presenting results by arms for multi‐arm clinical trials and lacking prospective evaluations (e.g. baseline or cross‐sectional evaluations of a clinical trial). Also excluded were papers reporting on opportunistic, unplanned ad‐hoc studies of clinical trials lacking a plausible hypothesis for an effect of the original intervention on health mediators or outcomes presented. A total of 27 manuscripts (9.4%) reported on prospective evaluations from NIH‐defined clinical trials and were included in the analytic database for detailed evaluation in wave 2.

#### 2.1.3 Phase III: Data abstraction and analysis

As indicated in the PRISMA 2020 Flow Diagram (Figure 1), our methodology using a grant to publication linkages identified a total of 76 publications for evaluation in wave 1 [37]. To optimize agreement and consistency among reviewers, a Data Capture Form (DCF) was developed and iteratively refined with reviewer feedback between rounds of review assignments. All eight reviewers were assigned to evaluate an equally divided compendium subset of all publications using the DCF, then evaluated half of another reviewer's compendium in addition to re‐reviewing half of their original compendium, and results were compared. The 27 publications in wave 2 were each independently evaluated by two reviewers. Any discrepancies or disagreements were resolved through discussions among reviewers.

The data abstracted and qualitatively analysed included types of study designs [38], geographic locations where the research took place, demographic characteristics, such as HIV status and age, mode of transmission and type of intervention, as well as whether major research aims addressed specific HPCC outcomes. These include discrete outcomes which span across the HIV prevention continuum and the HIV care continuum, and cross‐cutting outcomes defined by three domains: (1) sexual and reproductive health; (2) mental health and substance use; and (3) structural determinants of health (Table 1). Secular trends in grantee publications from NIH‐defined clinical trials linked to the grant portfolio before (wave 1) and after the PATC3H initiative (wave 2) were explored. Questions on the inclusion of age categories, key populations [39], and modes of transmission did not require mutually exclusive answers. A given answer choice was selected if, in the reviewer's expert assessment, that choice reflected most of the publication's research focus.

**Table 1:** Key definitions

**HIV prevention continuum**	**HIV care continuum**
HIV testing uptake	HIV diagnosis
Condom use	Linkage to care
PrEP uptake/use	Engagement in care
Microbicide uptake/use	Retention in care
VMMC uptake	ART initiation
ART as prevention use	ART adherence
Clean needles for PWID	Viral suppression
Cash transfer	Secondary outcomes

**Crosscutting outcomes**	
**Domains**	**Outcomes**
Sexual and reproductive health (SRH)	
	Access to SRH services, including consent in minors
	Condom use
	Sex work
	Sexual risk behaviours
	STI screening/treatment
Mental health and substance use (MHSU)	
	Adherence
	Drug use and treatment, including opioid substitution
	Mental illness and treatment
Structural determinants of health (SDOH)	
	Housing/Food insecurity
	Other^a^

Abbreviations: ART, antiretroviral therapy; PrEP, pre‐exposure prophylaxis; PWID, people who inject drugs; STI, sexually transmitted infection; VMMC, voluntary medical male circumcision.

^a^ Other includes stigma, disclosure, health service challenges, social support, social harms and structural problems not related to housing or food security.

## 3 RESULTS

### 3.1 Research landscape publication portfolio

Among all applications, 14% were funded and linked to 76 publications in wave 1, which included a variety of study designs (Table 2). Each of these studies substantially focused on AYA populations, HIV prevention and care, and cross‐cutting areas along the HPCC, spanning interventional, non‐interventional and non‐clinical research. Cross‐sectional, qualitative and cohort‐based studies made up the majority (66%), followed by randomized controlled clinical trial (20%) study designs.

**Table 2:** Research landscape publication portfolio characteristics

					Publications *N* = 76	(%)
**Age groups** ^a^						
<18 Years					62	82%
18–24 Years					43	57%
25 Years or more					18	24%
Age groups unknown/unclear					4	5%
All age groups included					8	11%
**Key populations** ^a^						
Men who have sex with men (MSM)					3	4%
Sex workers					3	4%
Drug users					2	3%
No key populations included					70	92%
**HIV status**						
At risk, uninfected					15	20%
At risk, uninfected and living with HIV					16	21%
Living with HIV					31	41%
HIV status unknown/unclear					14	18%
**Mode of HIV transmission** ^a^						
Behavioural (sexual)					37	49%
Behavioural (injecting drugs)					1	1%
Perinatal (mother‐to‐child)					21	28%
Other/unknown					25	33%
**Study design**						
Case control study					1	1%
Cohort study, retrospective					2	3%
Systematic review					2	3%
Ecological study					3	4%
Ideas, editorials and opinions					3	4%
Randomized controlled trial at the individual level					7	9%
Randomized controlled trial at the community level					8	11%
Other (pilot study, mixed methods, review article)					11	14%
Cohort study, prospective					12	16%
Qualitative interview: single or group administration					13	17%
Cross‐sectional study					14	18%

	Outcomes					
	Continuum of care *N* = 76	(%)	Crosscutting *N* = 79	(%)		
**Overall distribution**						
Continuum of care	44	58%			29	38%
Continuum of care and crosscutting	32	42%	25	32%	13	17%
Crosscutting			54	68%	34	45%
**Continuum of care**						
HIV prevention	17	22%			9	12%
HIV care	46	61%			28	37%
HIV prevention and care	13	17%			5	7%
Not targeted					34	45%
**Crosscutting domains**						
Sexual and reproductive health			19	24%	16	21%
Mental health and substance use			4	5%	4	5%
Structural determinants			17	22%	13	17%
Combination of domains			39	49%	14	18%
Not targeted					29	38%

^a^ Row percentages might not sum to 100% because categories did not have mutually exclusive representation.

**Figure 2:**
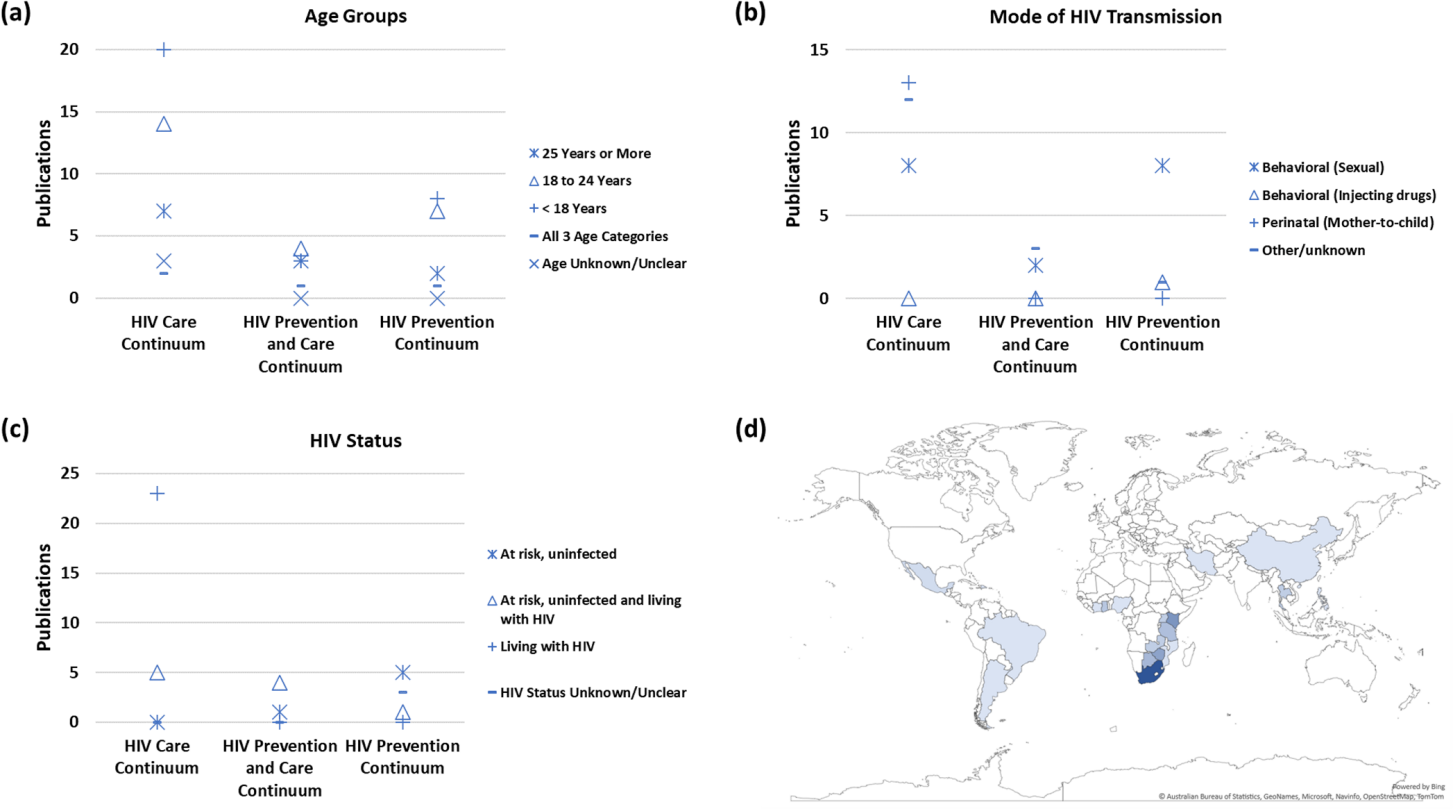
Publications in the landscape analysis (wave 1) which included milestones across HIV prevention and care continuum (HPCC) (*n* = 42) by adolescent and young adult (AYA) (a) age group, (b) mode of HIV transmission and (c) HIV status. The map in panel (d) shows the geographic representation of 70 publications that included country information. The colour intensity represents the number of publications that included a given country increasing from light to dark representing one up to 21 publications.

The participant populations targeted in these studies varied widely and representation within categories was not mutually exclusive (Table 2 and Figure 2). Eighteen publications included participants 25 years or older, 43 included AYA aged 18–24 and 62 included AYA less than 18 years of age. Most publications (70 [92%]) had no key population focus; among those that did, three addressed men who have sex with men, three addressed sex workers, two addressed drug users and none addressed prisoner or transgender populations. Most focused on AYA with HIV (*n* = 31), while similar numbers focused only on HIV‐uninfected AYA (*n* = 15), AYA with and without HIV (*n* = 16) and AYA whose status was unclear or unknown (*n* = 14). Mode of HIV transmission included behavioural (sexual, *n* = 37), perinatal (mother‐to‐child, *n* = 21) and behavioural (injection drug use, *n* = 1). The top five countries with the greatest representation include South Africa (*n* = 25), Kenya (*n* = 12), Zimbabwe (*n* = 10), Botswana (*n* = 8) and Tanzania (*n* = 6) (Figure 2).

### 3.2 Research landscape portfolio health outcomes representation across the HIV prevention and care continua

**Figure 3:**
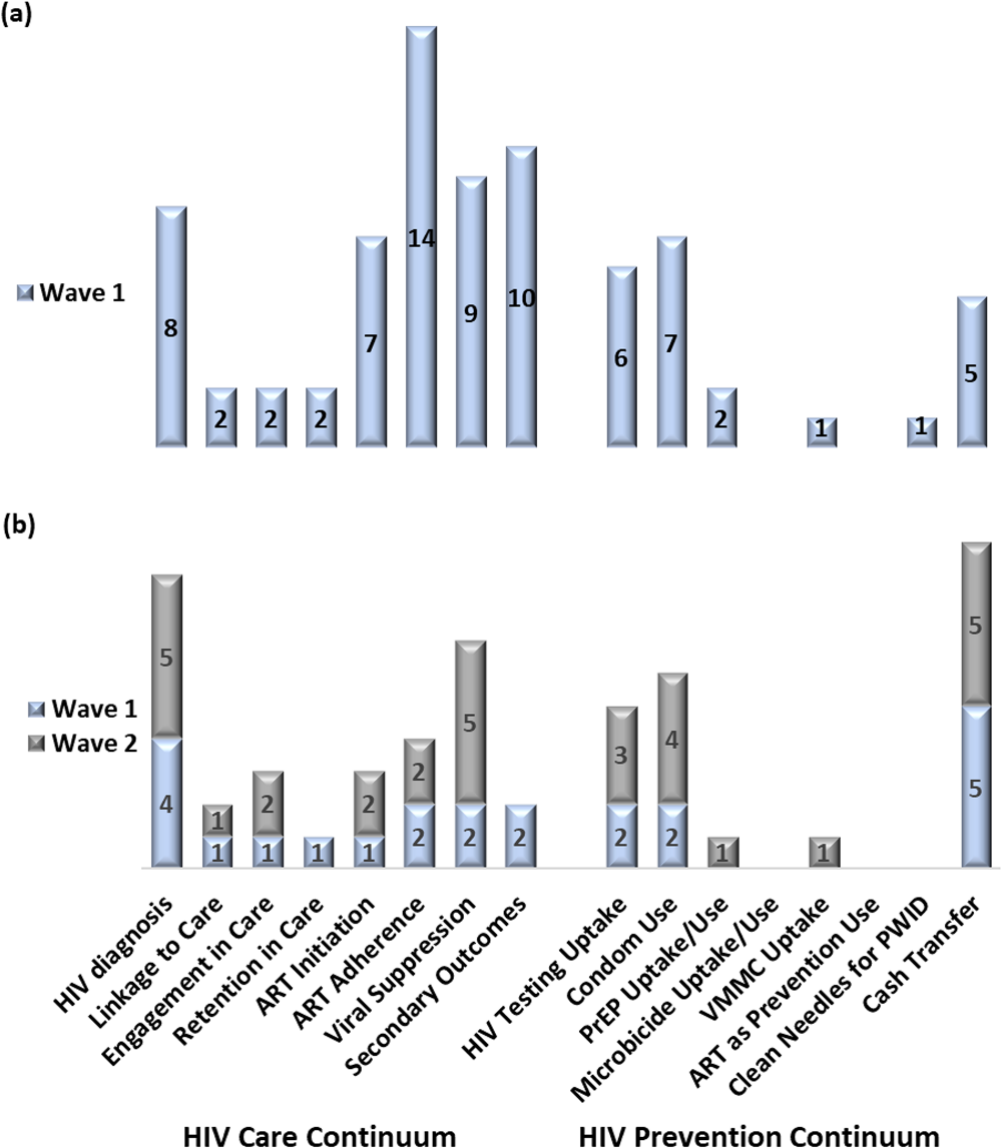
The adolescent and young adult (AYA) HIV prevention and care continuum (HPCC) in publications from (a) wave 1 landscape assessment among 42 publications that addressed 76 HPCC milestones, and (b) waves 1 and 2 NIH‐defined clinical trial evaluation among 30 publications that addressed 54 HPCC milestones.

The 76 publications in wave 1 revealed studies variably targeting milestones on the HPCC (Table 2), with some areas represented more frequently than others (Figure 3a). Among the 42 (55%) publications targeting discrete outcomes on the HPCC, nine (12%), 28 (37%) and five (7%) focused on the HIV prevention continuum, HIV care continuum and both, respectively (Table 2), while 47 (62%) focused on cross‐cutting outcome domains along the entire HPCC. Among 33 publications that included 54 HIV care continuum milestones, 48 (89%) milestones were on HIV diagnosis, ART initiation and adherence, viral suppression, and secondary outcomes, while 18 (82%) of 22 milestones in 14 publications that included HIV prevention continuum milestones were mainly on HIV testing, condom use and cash transfer interventions (Figure 3a). Among the 47 publications examining 79 cross‐cutting outcomes, 19 (24%), four (5%), 17 (22%), and 39 (49%) outcomes were focused on sexual and reproductive health, mental health and substance use, structural determinants of health or a combination of these domains, respectively. Gaps in milestones targeted on the HIV care continuum included linkage to, engagement and retention in care, and along the HIV prevention continuum included microbicide and voluntary male medical circumcision (VMMC) uptake, ART as prevention and use of clean needles for injection drug use.

### 3.3 Publications on NIH‐defined clinical trials across the HPCC

#### 3.3.1 Wave 1

Among the 76 publications from 2012 to 2017, 15 (20%) described AYA HPCC outcomes in an NIH‐defined clinical trial (Table S1). Interventions were categorized as behavioural, biomedical and the combination of both in 11 (73%), one (7%) and three (20%) of clinical trials, respectively. Among these, 10 (67%) targeted outcomes on the HPCC, with four (27%), three (20%) and three (20%) focused on the HIV prevention continuum, HIV care continuum and both, respectively (Table 3), while 13 (87%) focused on cross‐cutting outcome domains along the entire HPCC. In six publications that included 14 HIV care continuum milestones, 11 (79%) milestones were on HIV diagnosis, ART initiation and adherence, viral suppression, and secondary outcomes, while all nine milestones in seven publications that included HIV prevention continuum milestones were mainly on HIV testing, condom use and cash transfer interventions (Figure 3b). Among the 13 publications addressing 24 crosscutting outcomes, six (25%), one (4%), three (13%) and 14 (58%) outcomes targeted sexual and reproductive health, mental health and substance use, structural determinants of health or a combination of these domains, respectively (Table 3). Overall, there was relatively little to no emphasis on structural issues like housing and food security, sexually transmitted infection (STI) screening or sex work (Figure S1a), and no research publications on ethics or regulatory aspects of the research were identified. Of those that measured the relationship of interventions to outcomes, eight (53%) studies reported a statistically significant relationship between a given intervention's effect on at least one outcome.

**Table 3:** US National Institutes of Health‐defined clinical trial publication portfolio characteristics

									Publications			
									Wave 1, *N* = 15	(%)	Wave 2, *N* = 27	(%)
**Age groups^a^**												
< 18 Years									11	73%	21	78%
18–24 Years									10	67%	15	56%
25 Years or more									4	27%	4	15%
All age groups included									1	7%	–	–
**Key populations^a^**												
Men who have sex with men (MSM)									1	7%	–	–
Sex workers									2	13%	2	7%
Drug users									–	–	2	7%
No key populations included									13	87%	23	85%
**HIV status**												
At risk, uninfected									5	33%	9	33%
At risk, uninfected and living with HIV									3	20%	1	4%
Living with HIV									4	27%	11	41%
HIV status unknown/unclear									3	20%	6	22%
**Mode of HIV transmission^a^**												
Behavioural (sexual)									9	60%	4	15%
Behavioural (injecting drugs)									–	–	–	–
Perinatal (mother‐to‐child)									2	13%	2	7%
Other/unknown									4	27%	21	78%

	Outcomes											
	Continuum of care				Crosscutting							
	Wave 1, *N* = 23	(%)	Wave 2, *N* = 31	(%)	Wave 1, *N* = 24	(%)	Wave 2, *N* = 30	(%)				
**Overall distribution**												
Continuum of care	2	9%	16	52%					2	13%	11	41%
Continuum of care and crosscutting	21	91%	15	48%	17	71%	16	53%	8	53%	9	33%
Crosscutting					7	29%	14	47%	5	33%	7	26%
**Continuum of care**												
HIV prevention	5	22%	10	32%					4	27%	8	30%
HIV care	8	35%	12	39%					3	20%	10	37%
HIV prevention and care	10	43%	9	29%					3	20%	2	7%
Not targeted									5	33%	7	26%
**Crosscutting domains**												
Sexual and reproductive health					6	25%	3	10%	5	33%	3	11%
Mental health and substance use					1	4%	4	13%	1	7%	4	15%
Structural determinants					3	13%	2	7%	3	20%	2	7%
Combination of domains					14	58%	21	70%	4	27%	7	26%
Not targeted									2	13%	11	41%

^a^Row percentages might not sum to 100% because categories did not have mutually exclusive representation.

#### 3.3.2 Wave 2

All 27 publications from 2018 to 2021 described HPCC outcomes from clinical trials in AYA (Table S2). Interventions were categorized as behavioural, biomedical and a combination of both in 22 (82%), four (15%) and one (4%), respectively. Among these, 20 (74%) targeted outcomes on the HPCC, and eight (30%), 10 (37%) and two (7%) focused on the HIV prevention continuum, HIV care continuum and both, respectively (Table 3), while 16 (59%) focused on cross‐cutting outcome domains along the entire HPCC. In 12 publications that included 17 HIV care continuum milestones, 14 (82%) milestones were on HIV diagnosis, ART initiation and adherence, and viral suppression (Figure 3b). In contrast to wave 1 clinical trials, there were two milestones on engagement in care but none on secondary outcomes. Like wave 1, 12 (86%) of the 14 milestones in 10 publications that included HIV prevention continuum milestones targeted HIV testing, condom use and cash transfer interventions (Figure 3b). Among the 16 (59%) publications addressing 30 crosscutting outcomes, three (10%), four (13%), two (7%) and 21 (70%) outcomes targeted sexual and reproductive health, mental health and substance use, structural determinants of health or a combination of these domains, respectively (Table 3). There was some added emphasis on condom use, STI screening, sex work and drug use treatment outcomes (Figure S1b) compared to wave 1. Of those that measured the relationship of interventions to outcomes, 21 of the studies reported a statistically significant relationship between a given intervention's effect on at least one outcome.

### 3.4 Improving trends but gaps on the AYA HPCC remain

HIV prevention, care and treatment research include discrete and cross‐cutting topics that encompass epidemiology, diagnosis, clinical manifestations, pathogenesis, transmission, treatment and prevention. This targeted review examined and coded the NIH grant portfolio to characterize the landscape for AYA HIV research in resource‐limited settings. Overall, the manuscript study design types and populations of focus across the continua determined by reviewers matched well with the empiric coding of the grant portfolio. While in the broader research landscape analysis (wave 1), there was a much higher proportion (65 [43%]) of the 151 linked publications that lacked either substantial focus on the population or outcomes of interest and were excluded, limiting the scope to NIH‐defined clinical trials in wave 2 greatly improved this focus with only four (1%) of the 286 linked publications being excluded (Figure 1). However, similar proportions of NIH‐defined clinical trials, 15 (10%) and 27 (9%), were identified in the first and second waves, respectively. The representation of demographic and clinical characteristics was also similar in publications between the two waves (Tables 2 and 3). Though, the density of geographic overrepresentation in SSA (Figure 2) and paucity in key populations was notable.

**Figure 4:**
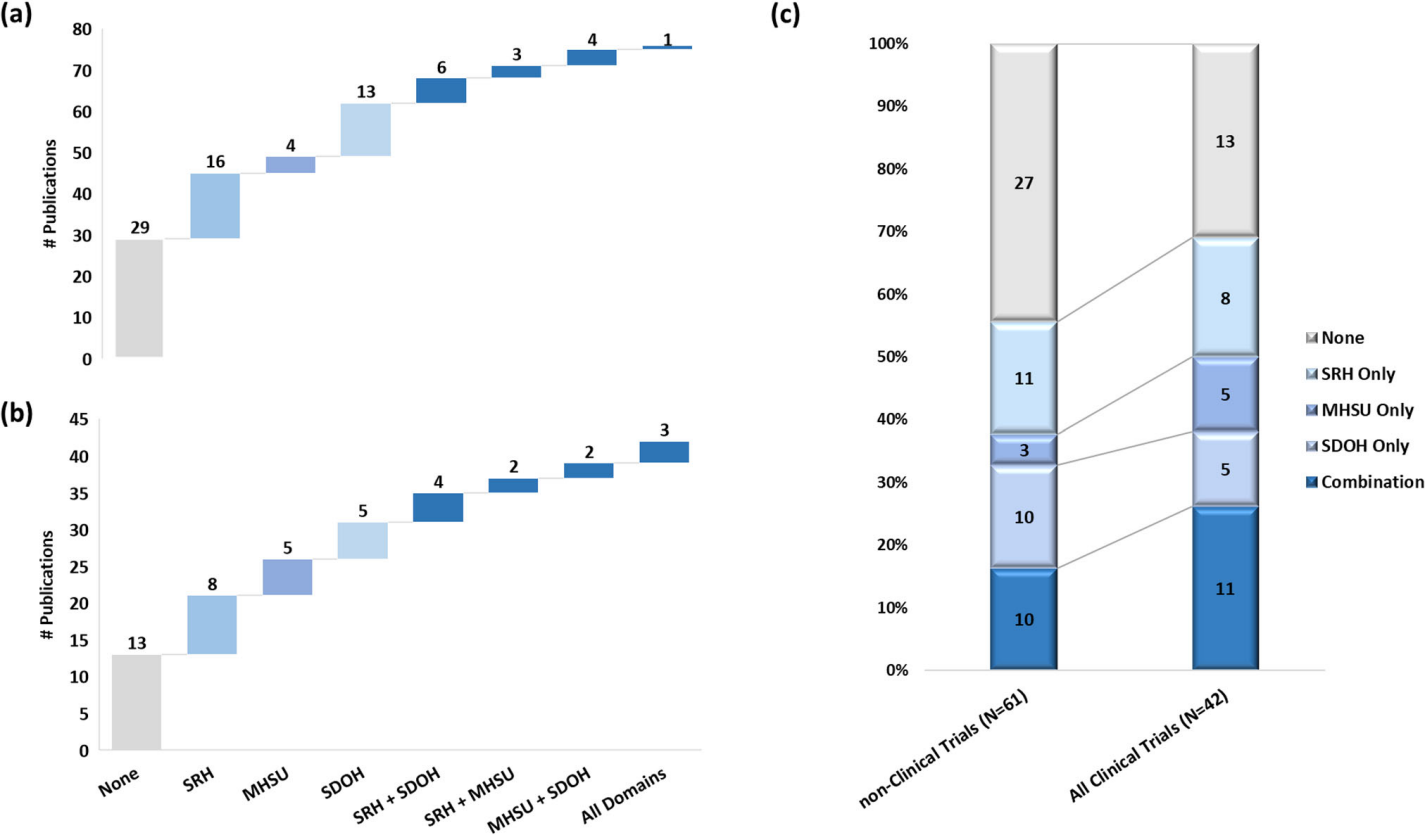
The frequency distribution of and gaps in cross‐cutting domains among publications from 2012 to 2021 in the (a) research landscape assessment (w*ave 1*: 79 outcomes in 47 publications), and (b) US National Institutes of Health‐defined clinical trial evaluation (*waves 1 and 2*: 54 outcomes in 29 publications). Proportional representation of crosscutting domains (c) in publications of non‐clinical trials *(subset of wave 1 without clinical trials)* compared to all clinical trials (*waves 1 and 2*). Domain definitions are SRH: sexual and reproductive health, including access to SRH services, such as consent among minors, condom use, sex work, sexual risk behaviours, and sexually transmitted infection (STI) screening and treatment; MHSU: mental health and substance use, including adherence, drug use and treatment, such as opioid substitution, and mental illness and treatment; SDOH: structural determinants of health, including housing and food security, and other structural determinants, such as stigma, disclosure, health service challenges, social support, social harms and structural problems not related to housing or food security.

Incremental improvement in the representation of major milestones on the AYA‐specific HPCC (Figure 3b) and crosscutting outcome domains (Figure 4) was noted in publications from wave 1 (2012–2017) compared to wave 2 (2018–2021). However, significant gaps remain for NIH‐defined clinical trials on the HIV care continuum for access to and retention in HIV care and were particularly striking on the HIV prevention continuum for the uptake of newer biomedical prevention modalities (Figure 3b). Only two (5%) of the 42 clinical trial publications addressed prevention: one each on VMMC and oral pre‐exposure prophylaxis uptake, respectively. Taken together with the predominating focus on HIV testing, condoms and conditional cash transfers in the HIV prevention continuum reflects the maturing trajectory of the HIV prevention field during this period. In exploring trends of crosscutting outcomes, no differences were noted between domains represented in publications when comparing wave 1 to 2 (Figure 4a,b). However, the 42 publications on clinical trials showed proportionately more focus on domains of mental health and substance use, and its combination with sexual and reproductive health and/or structural determinants of health, and similar emphasis on sexual and reproductive health alone, compared to a subset of 61 publications without clinical trials from wave 1, and there were fewer publications without any domain represented (Figure 4c).

## 4 DISCUSSION

In this systematic but targeted analysis of NIH‐supported research and result dissemination, modest progress has been demonstrated in clinical trials across the HPCC for AYA affected by HIV in LMIC. However, substantial unmet needs were noted on access to and retention in HIV care for AYA with HIV and on the uptake of novel biomedical interventions for both treatment and prevention of HIV. The enhanced attention to critical crosscutting issues within the domains of sexual and reproductive health, mental health and substance use, and structural determinants of health is encouraging as they underpin the successful achievement of milestones across the AYA HPCC. However, despite the added focus on condom use, STI screening and sexual risk behaviours, sex work and drug use treatment outcomes in clinical trials (Figure S1b), these areas need continued attention. Also, added emphasis must be placed on structural issues like housing and food security, and on improving access to sexual and reproductive health services. Our results are consistent with findings from the only contemporaneous systematic review of HIV service programming across the AYA HPCC commissioned by the United Nations International Children's Emergency Fund (UNICEF) [40]. While the authors demonstrated several consistently effective high‐quality interventions in adults like VMMC, ART and condoms, there was a paucity of adolescent‐specific studies to inform policy decisions on the uptake of and access and adherence to these among AYA. Fundamental to improving access to these services for minors are contextually appropriate ethical, legal and regulatory frameworks to guide such efforts. However, no publications on empiric ethical or regulatory research were identified, despite that the majority of incident HIV infections in SSA are among adolescent girls and young women [31, 41]. Understanding the relationship between a grant portfolio and its corresponding publications is one of many evaluable benchmarks of productivity, visibility and influence in the field of AYA HIV prevention and care. Such metrics not only can yield valuable information about the return on investment, but more importantly, can inform future directions and focus resources.

It is essential that individuals receive and engage in regular medical care to reap the health benefits of ART and sustained viral suppression, including the prevention of onward transmission. Additionally, the HIV prevention continuum's overall goals include ensuring an individual remains HIV uninfected through the receipt of and engagement in services to prevent HIV acquisition. Without an individual engaging and sustaining in the entirety of the HPCC, partial engagement at various milestones impedes the overall goal of ending new HIV infections. Our results show many gaps along the HPCC, several of which are surmountable with additional resources and focus. Clinical trials have been widely used to robustly determine the effectiveness of interventions in other contexts as in adults with chronic illnesses like HIV, yet they remain disproportionately low among clinical research in AYA, especially in key populations and throughout many LMICs outside of SSA. Unique developmental and cultural needs among AYA populations affected by HIV suggest that a one‐size‐fits‐all approach is insufficient and argue that differentiated care models are important to address the needs of AYA rigorously and comprehensively and improve their HPCC health outcomes.

### 4.1 Limitations of present review

This review reflects only grants and linked publications receiving support from the NIH limiting the scope of work identified and in the field to this subset of projects. It was not feasible to analyse grants such as cooperative agreements supporting large HIV clinical trial networks by this approach and those are not reported in this review. Furthermore, the inclusion of multiple, often overlapping, and non‐disaggregated age groups across the lifespan in published studies limited our ability to search grants based on distinct age ranges, requiring the use of additional descriptive terms like “adolescents” and/or “young adults.” As the focus of this review was confined to LMIC, it does not capture the ongoing efforts in large networks and/or programmes in the United States, and limits generalizability to all AYA. While this review included publications as recent as 2021, they were linked to grants funded up to 2017 and more awards may have been issued since. Lastly, it is important to note that ongoing grants, especially those recently awarded, are likely to result in additional publications and should be re‐evaluated at a later date.

## 5 CONCLUSIONS

As the first detailed review of a US NIH‐supported portfolio specific to international AYA HIV research, this paper provides the foundation for future discussions and collaborations to improve the health of AYA at risk of or with HIV globally. Although it is encouraging to see ongoing NIH activities and data with incremental advances towards expected trends, including a greater focus on the HPCC, there is a critical need for more research and innovation on access to and retention in care on the HIV care continuum, and uptake of novel biomedical prevention and treatment interventions on the entire HPCC. Added attention must be given to cross‐cutting structural issues like housing and food security, and on improving access to sexual and reproductive health services. Foundational to the success of these efforts will be increased empirical research to inform ethical, legal and regulatory frameworks to enhance the inclusion of adolescents in HIV research and clinical trials. Reviews of this nature should continue to be explored and strengthened to better understand the current and future trajectories of the NIH's investment and alignment with international HIV health priorities.

## COMPETING INTERESTS

SC, MP, SL, SA, PJP, NC, JM and BGK disclose no competing interests. As an employee of the NIH Office of AIDS Research during this analysis, GB had no competing interests. However, GB joined Gilead Sciences in May 2018, an organization that did not provide funding for this study but may have a financial or non‐financial interest in the subject matter or materials discussed in this manuscript.

## AUTHORS’ CONTRIBUTIONS

SC conceptualized and designed the methodology, performed data curation and analysis, and drafted the manuscript. MP conceptualized and designed the methodology, supervised data curation and critically revised the manuscript for important intellectual content. SA performed data curation and analysis, and critically revised the manuscript for important intellectual content. GB performed data curation and analysis, and critically revised the manuscript for important intellectual content. PJP performed data curation and analysis, and critically revised the manuscript for important intellectual content. SL performed data curation and analysis, and critically revised the manuscript for important intellectual content. NC performed data curation and analysis, and critically revised the manuscript for important intellectual content. JM performed data curation and analysis, and critically revised the manuscript for important intellectual content. BGK conceptualized and designed the methodology, supervised data curation and analysis, and critically reviewed and revised the manuscript for important intellectual content.

## DISCLAIMER

The content is solely the responsibility of the authors and does not necessarily reflect the official views of the Department of Health and Human Services or the National Institutes of Health.

## Supporting information


**Figure S1**: The frequency distribution of and gaps in cross‐cutting outcomes among publications from 2012 to 2021 in the (**a**) research landscape assessment (*wave 1*: 79 outcomes in 47 publications), and (**b**) NIH‐defined clinical trial evaluation (*waves 1 and 2*: 54 outcomes in 29 publications). STI: sexually transmitted infection, SRHS: sexual and reproductive health services, Other: structural determinants such as stigma, disclosure, health service challenges, social support, social harms, and structural problems not related to housing or food security.

**Table S1**: Publications of US National Institutes Health‐Defined Clinical Trials on the Adolescent and Young Adult HIV Prevention and/or Care Continuum (HPCC) from Wave 1, 2012–2017 (N=15)

**Table S2**: Publications of US National Institutes Health‐Defined Clinical Trials on the Adolescent and Young Adult HIV Prevention and/or Care Continuum (HPCC) from Wave 2, 2018–2021 (N=27)

**File S1**: A Comprehensive Care Continuum for HIV‐affected Adolescents in Resource Constrained Settings (PATC^3^H)

## Data Availability

Research data are not shared.
