# Supplementary figures and images for: Adolescent and young adult research across the HIV prevention and care continua: an international programme analysis and targeted review
Source: J Int AIDS Soc. 2023 Mar 23;26(3):e26065. doi: 10.1002/jia2.26065 (PMC10034634; doi:10.1002/jia2.26065)

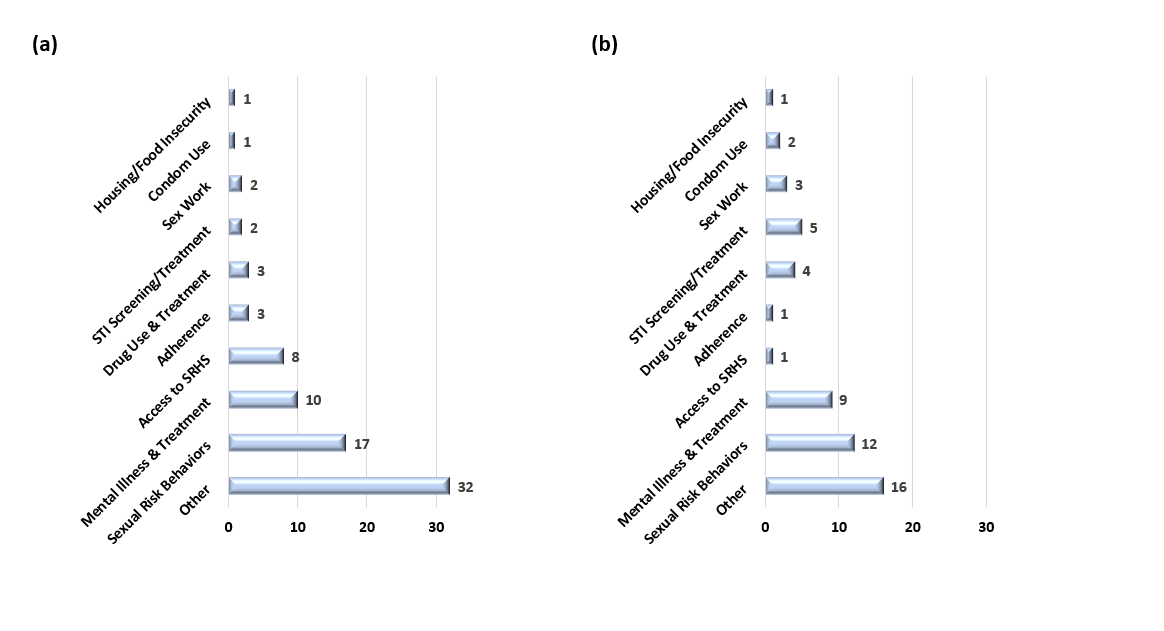

Supplement: Supplementary file 1 — Figure S1: The frequency distribution of and gaps in cross‐cutting outcomes among publications from 2012 to 2021 in the (a) research landscape assessment (wave 1: 79 outcomes in 47 publications), and (b) NIH‐defined clinical trial evaluation (waves 1 and 2: 54 outcomes in 29 publications). STI: sexually transmitted infection, SRHS: sexual and reproductive health services, Other: structural determinants such as stigma, disclosure, health service challenges, social support, social harms, and structural problems not related to housing or food security. [file JIA2-26-e26065-s002.docx]
