# Supplementary material for: Adolescent and young adult research across the HIV prevention and care continua: an international programme analysis and targeted review
Source: J Int AIDS Soc. 2023 Mar 23;26(3):e26065. doi: 10.1002/jia2.26065 (PMC10034634; doi:10.1002/jia2.26065)
Supplement: Supplementary file 2 — Table S1: Publications of US National Institutes Health‐Defined Clinical Trials on the Adolescent and Young Adult HIV Prevention and/or Care Continuum (HPCC) from Wave 1, 2012–2017 (N=15) [file JIA2-26-e26065-s004.docx]

| **Supplementary Table 1: Publications of US National Institutes Health-Defined Clinical Trials on the Adolescent and Young Adult HIV Prevention and/or Care Continuum (HPCC) from Wave 1, 2012-2017 (N=15)** | | | | | | | |
| --- | --- | --- | --- | --- | --- | --- | --- |
|  | **First Author, Year** | **Randomization Level** | **Key Population Studied§** | **HIV Status and Age Range** | **Mode of Transmission** | **Outcome(s) of Interest** | **Intervention Type** |
|  | **PubMed ID** |  |  |  |  |  |  |
| **Positive Effect** | Miller, 2013 | Community | None | HIV (-), Uninfected, <18 Years | Behavioral (Sexual) | Quality Adjusted Life Years, Cost Effectiveness, Structural Problems (Early Marriage and School Retention) | Behavioral |
|  | PMID: 23334923 |  |  |  |  |  |  |
|  | Small, 2014 | Individual | None | HIV (+), Living with HIV, <18 Years | Perinatal (Mother-to-Child) | Mental Illness and Treatment (Child Emotional Distress) | Behavioral |
|  | PMID: 25364654 |  |  |  |  |  |  |
|  | Handa, 2014 | Community | None | HIV Status Unknown/Unclear, <18 Years, and 18 to 24 Years | Behavioral (Sexual) | Sexual Risk Behaviors (Sexual Debut) | Behavioral |
|  | PMID: 24454875 |  |  |  |  |  |  |
|  | Handa, 2015 | Community | None | HIV Status Unknown/Unclear, <18 Years, and 18 to 24 Years | Other/Unknown | Unconditional Cash Transfer (UCT), Early Pregnancy and Marriage | Behavioral |
|  | PMID: 26246032 |  |  |  |  |  |  |
|  | Hallfors, 2015 | Community | None | HIV (-), Uninfected, <18 Years | Other/Unknown | Schooling, School Drop Out, Sexual Debut, Marriage, Pregnancy, Structural Problems | Combined |
|  | PMID: 25530603 |  |  |  |  |  |  |
|  | Kilburn, 2016 | Community | None | HIV Status Unknown/Unclear, <18 Years, and 18 to 24 Years | Other/Unknown | Cash Transfer, Mental Health, Employment, School Enrollment | Behavioral |
|  | PMID: 26576822 |  |  |  |  |  |  |
|  | Pettifor, 2016 | Individual | None | HIV (-), Uninfected and HIV (+), Living with HIV, <18 Years, and 18 to 24 Years | Other/Unknown | Sexual Risk Behaviors, Transactional Sex, Violence, HIV Diagnosis, Cash Transfer, School Attendance, Secondary Outcomes (HSV Incidence) | Combined |
|  | PMID: 27815148 |  |  |  |  |  |  |
|  | Handa, 2017 | Individual | None | HIV (-), Uninfected, <18 Years, 18 to 24 Years, and 25 Years or more | Behavioral (Sexual) | Sexual Risk Behaviors, Sexual Debut | Behavioral |
|  | PMID: 26853950 |  |  |  |  |  |  |
| **No Effect** | Rosenberg, 2014 | Individual | None | HIV (-), Uninfected, <18 Years, and 18 to 24 Years | Behavioral (Sexual) | Sexual Risk Behaviors, Cash Transfer | Behavioral |
|  | PMID: 23612944 |  |  |  |  |  |  |
|  | Galárraga, 2014 | Individual | Men who have Sex with Men (MSM), Sex Workers | HIV (-), Uninfected and HIV (+), Living with HIV, 18 to 24 Years, and 25 Years or more | Behavioral (Sexual) | HIV Diagnosis, HIV Status, Sex Work, STI Screening/Treatment | Behavioral |
|  | PMID: 25399543 |  |  |  |  |  |  |
|  | Bhana, 2014 | Individual | None | HIV (+), Living with HIV, <18 Years | Perinatal (Mother-to-Child) | Adherence, Disclosure, Stigma, Mental Illness and Treatment | Behavioral |
|  | PMID: 23767772 |  |  |  |  |  |  |
|  | Rotheram-Borus, 2016 | Community | None | HIV (-), Uninfected, 18 to 24 Years, and 25 Years or more | Behavioral (Sexual) | Sexual Risk Behaviors (Sex Partners) HIV Testing, Substance Use, Condom Use | Behavioral |
|  | PMID: 26837624 |  |  |  |  |  |  |
|  | Bermudez, 2016 | Individual | None | HIV (+), Living with HIV, <18 Years | Behavioral (Sexual) | ART Adherence, Structural Problems (Household Assets, Cash Savings, Food Security etc.) | Behavioral |
|  | PMID: 27392003 |  |  |  |  |  |  |
|  | Kerrigan, 2017 | Community | Sex Workers | HIV (-), Uninfected and HIV (+), Living with HIV, 18 to 24 Years and 25 Years or more | Behavioral (Sexual) | HIV Testing Uptake, Engagement in Care, ART Adherence, Condom Use, Viral Suppression, | Combined |
|  | PMID: 27930613 |  |  |  |  |  |  |
|  | Kiene, 2017 | Community | None | HIV (+), Living with HIV, 18 to 24 Years and 25 Years or more | Behavioral (Sexual) | HIV Diagnosis, Linkage to Care, ART Initiation, Retention in Care, Viral Suppression, Cost Effectiveness | Biomedical |
|  | PMID: 28673251 |  |  |  |  |  |  |
|  | § Key Population Studied defined as those including Men who have Sex with Men (MSM), Gay Men, Prisoners, Sex Workers, and People Who Inject Drugs [39] | | | | | | |
