# Supplementary material for: Adolescent and young adult research across the HIV prevention and care continua: an international programme analysis and targeted review
Source: J Int AIDS Soc. 2023 Mar 23;26(3):e26065. doi: 10.1002/jia2.26065 (PMC10034634; doi:10.1002/jia2.26065)
Supplement: Supplementary file 3 — Table S2: Publications of US National Institutes Health‐Defined Clinical Trials on the Adolescent and Young Adult HIV Prevention and/or Care Continuum (HPCC) from Wave 2, 2018–2021 (N=27) [file JIA2-26-e26065-s001.docx]

| **Supplementary Table 2: Publications of US National Institutes Health-Defined Clinical Trials on the Adolescent and Young Adult HIV Prevention and/or Care Continuum (HPCC) from Wave 2, 2018-2021 (N=27)** | | | | | | | |
| --- | --- | --- | --- | --- | --- | --- | --- |
|  | **First Author, Year** | **Randomization Level** | **Key Population Studied§** | **HIV Status and Age Range** | **Mode of Transmission** | **Outcome(s) of Interest** | **Intervention Type** |
|  | **PubMed ID** |  |  |  |  |  |  |
| **Positive Effect** | Cho, 2018 | Community | None | HIV (-), Uninfected, <18 Years, and 18 to 24 Years | Other/Unknown | HIV Diagnosis, Condom Use, VMMC Uptake, Cash Transfer, Sexual Risk Behaviors, STI Screening/Treatment, Transactional Sex | Behavioral |
|  | PMID: 29107569 |  |  |  |  |  |  |
|  | Killburn, 2018 | Community | None | HIV (-), Uninfected, <18 Years, and 18 to 24 Years | Other/Unknown | Cash Transfer, Physical, Sexual Risk Behaviors, Sexual Debut | Behavioral |
|  | PMID: 29485746 |  |  |  |  |  |  |
|  | Pettifor, 2018 | Community | None | HIV (-), Uninfected, 18 to 24 Years, and 25 Years or more | Other/Unknown | Sexual Risk Behaviors, Condom Use, Drug Use & Treatment, Gender Norms, and Intimate Partner Violence | Behavioral |
|  | PMID: 29972287 |  |  |  |  |  |  |
|  | Ismayilova, 2018 | Individual | Drug Users | HIV Status Unknown/Unclear, <18 Years | Other/Unknown | Drug Use & Treatment, Substance Use including Opioid Substitution, Alcohol | Behavioral |
|  | PMID: 30006027 |  |  |  |  |  |  |
|  | Winskell, 2018 | Individual | None | HIV Status Unknown/Unclear, <18 Years | Other/Unknown | Condom Use | Behavioral |
|  | PMID: 30068501 |  |  |  |  |  |  |
|  | Green, 2019 | Community | None | HIV Status Unknown/Unclear, <18 Years | Other/Unknown | Cash Transfer, Major Depression | Behavioral |
|  | PMID: 30055002 |  |  |  |  |  |  |
|  | Kilburn, 2019 | Community | None | HIV (-), Uninfected, <18 Years, and 18 to 24 Years | Other/Unknown | Cash Transfer, Sexual Risk Behaviors, Mental Illness & Treatment, Sexual Relationship Power | Behavioral |
|  | PMID: 30415429 |  |  |  |  |  |  |
|  | Nestadt, 2019 | Individual | None | HIV (+), Living with HIV, <18 Years | Perinatal (Mother-to-Child) | ART Adherence, Mental Illness and Treatment, | Behavioral |
|  | PMID: 31067121 |  |  |  |  |  |  |
|  | Kerrigan, 2019 | Community | Sex Workers | HIV (-), Uninfected and HIV (+), Living with HIV, 18 to 24 Years and 25 Years or more | Behavioral (Sexual) | HIV Diagnosis, Engagement in Care, ART Initiation, Viral Suppression, Condom Use, | Behavioral |
|  | PMID: 31513552 |  |  |  |  |  |  |
|  | Wilson, 2019 | Individual | None | HIV (+), Living with HIV, <18 Years, and 18 to 24 Years | Other/Unknown | Engagement in Care | Behavioral |
|  | PMID: 30932957 |  |  |  |  |  |  |
|  | Ssewamala, 2020 | Community | None | HIV (+), Living with HIV, <18 Years | Other/Unknown | Viral Suppression | Behavioral |
|  | PMID: 32040523 |  |  |  |  |  |  |
|  | Kuo, 2020 | Community | None | HIV Status Unknown/Unclear, <18 Years | Behavioral (Sexual) | Sexual Risk Behaviors, Condom Use, Mental Illness & Treatment, Resilience | Behavioral |
|  | PMID: 32202920 |  |  |  |  |  |  |
|  | Dow, 2020 | Individual | None | HIV (+), Living with HIV, <18 Years, and 18 to 24 Years | Perinatal (Mother-to-Child) | ART Adherence, Viral Suppression, Mental Illness & Treatment, | Behavioral |
|  | PMID: 32887558 |  |  |  |  |  |  |
|  | Konstantinus, 2020 | Individual | None | HIV (-), Uninfected, <18 Years, and 18 to 24 Years | Other/Unknown | HIV Diagnosis | Biomedical |
|  | PMID: 31675420 |  |  |  |  |  |  |
|  | Pettifor, 2020 | Individual | None | HIV Status Unknown/Unclear, 18 to 24 Years | Behavioral (Sexual) | HIV Testing Uptake, Peer and Sex Partner Reporting of HIV Testing | Biomedical |
|  | PMID: 32322811 |  |  |  |  |  |  |
|  | Balle, 2020 | Individual | None | HIV (-), Uninfected, <18 Years, and 18 to 24 Years | Other/Unknown | HIV Diagnosis | Biomedical |
|  | PMID: 33149114 |  |  |  |  |  |  |
|  | Gill, 2020 | Other Intervention, Not Randomized or Controlled | None | HIV (-), Uninfected, <18 Years, and 18 to 24 Years | Other/Unknown | HIV Testing Uptake, PrEP Uptake/Use, STI Screening/Treatment | Combined |
|  | PMID: 33222803 |  |  |  |  |  |  |
|  | Cavazos-Rehg, 2021 | Community | None | HIV (+), Living with HIV, <18 Years | Other/Unknown | Mental Illness & Treatment, Hopelessness, Depression | Behavioral |
|  | PMID: 32980245 |  |  |  |  |  |  |
|  | Ybarra, 2021 | Individual | None | HIV (-), Uninfected, 18 to 24 Years | Behavioral (Sexual) | HIV Testing Uptake, Condom Use, Abstaining from Sex | Behavioral |
|  | PMID: 33963477 |  |  |  |  |  |  |
|  | Cavazos-Rehg, 2021 | Community | None | HIV (+), Living with HIV, <18 Years | Other/Unknown | Mental Illness & Treatment, Depression, Hopelessness | Behavioral |
|  | PMID: 34020312 |  |  |  |  |  |  |
|  | Tozan, 2021 | Community | None | HIV (+), Living with HIV, <18 Years | Other/Unknown | Viral Suppression | Behavioral |
|  | PMID: 34176245 |  |  |  |  |  |  |
| **No Effect** | Maughan-Brown, 2018 | Individual | None | HIV (+), Living with HIV, <18 Years, 18 to 24 Years, and 25 Years or more | Other/Unknown | Linkage to Care, ART Initiation | Behavioral |
|  | PMID: 29432045 |  |  |  |  |  |  |
|  | Bermudez, 2018 | Community | None | HIV (+), Living with HIV, <18 Years | Other/Unknown | Viral Suppression | Behavioral |
|  | PMID: 29846836 |  |  |  |  |  |  |
|  | Ismayilova, 2019 | Individual | Drug Users | HIV Status Unknown/Unclear, <18 Years | Other/Unknown | Sexual Risk Behaviors, Risk Behavior Communication, Drug Use & Treatment | Behavioral |
|  | PMID: 31003194 |  |  |  |  |  |  |
|  | Balle, 2021 | Individual | None | HIV (-), Uninfected, <18 Years, and 18 to 24 Years | Other/Unknown | HIV Diagnosis, STI Screening/Treatment, Contraceptives | Biomedical |
|  | PMID: 32989170 |  |  |  |  |  |  |
|  | Shato, 2021 | Community | None | HIV (+), Living with HIV, <18 Years | Other/Unknown | Cash Transfer, | Behavioral |
|  | PMID: 33812750 |  |  |  |  |  |  |
|  | Beckham, 2021 | Individual | Sex Workers | HIV (+), Living with HIV, 18 to 24 Years, and 25 Years or more | Other/Unknown | Access to Sexual Reproductive Health Services | Behavioral |
|  | PMID: 34247614 |  |  |  |  |  |  |
|  | § Key Population Studied defined as those including Men who have Sex with Men (MSM), Gay Men, Prisoners, Sex Workers, and People Who Inject Drugs [39] | | | | | | |
