# Supplementary material for: Adolescent and young adult research across the HIV prevention and care continua: an international programme analysis and targeted review
Source: J Int AIDS Soc. 2023 Mar 23;26(3):e26065. doi: 10.1002/jia2.26065 (PMC10034634; doi:10.1002/jia2.26065)
Supplement: Supplementary file 4 — File S1: A Comprehensive Care Continuum for HIV‐affected Adolescents in Resource Constrained Settings (PATC3H) [file JIA2-26-e26065-s003.docx]

**A Comprehensive Care Continuum for HIV-affected Adolescents in Resource Constrained Settings (PATC^3^H)**

The data gathered from this targeted review informed a new NIH phased-innovation initiative for an international research program to prevent and treat HIV infection among adolescents and young adults in seven African countries and Brazil called the Prevention and Treatment through a Comprehensive Care Continuum for HIV-affected Adolescents in Resource Constrained Settings (PATC^3^H) ([RFA-HD-18-032](http://grants1.nih.gov/grants/guide/rfa-files/RFA-HD-18-032.html)). These projects are phased into large scale evaluation of effectiveness after an initial formative study period demonstrates feasibility and preliminary effectiveness through successful achievement of a priori defined benchmarks.

The PATC^3^H research program, through collaborations with the National Institute of Minority Health and Health Disparities (NIMHD), the Office of Behavioral and Social Sciences Research (OBSSR), and the Office of AIDS Research (OAR), supports the development of 8 strategies to identify and reach youth at-risk of HIV infection and those living with HIV to enroll into medical care programs. Collaborations amongst the 8 programs is encouraged to refine and strengthen each individual program to address gaps across the HPCC.

Of the eight recently awarded PATC^3^H grants there is an even distribution of programs along the continuum; three on HIV treatment, three on HIV prevention, and two on both HIV prevention and treatment. The eight awards capture adolescents, both at risk and those living with HIV, ranging from 10 to 24 years old, and includes the key populations [29] of young transgender women and men who have sex with men. Focus was placed on testing frequency, uptake, and behavior to address HIV prevention as well as digital health interventions and data informed stepped care, among other strategies to address HIV care. Other unifying themes and priorities include youth engagement, addressing health disparities, and overall improvements in HIV testing and/or linkage to care across service delivery systems of the HPCC. This consortium will employ clinical research, implementation science, and evaluation across the individual, family, community, structural and education, and health systems levels to address gaps identified by this program analysis specific to the HPCC. A list of the PATC^3^H Programs and Principal Investigators (PIs) can be found in the Table.

**Table: PATC^3^H Programs and Principal Investigators**

| **Project Title (Linked to NIH RePORTER)** | **PI Name** | **Country** |
| --- | --- | --- |
| [Strategic antiretroviral therapy and HIV testing for youth in rural Africa (SEARCH-Youth)](https://projectreporter.nih.gov/project_info_description.cfm?aid=9618066) | Diane Havlir | Kenya & Uganda |
| [Data-informed Stepped Care (DiSC) to Improve Adolescent HIV Outcomes](https://projectreporter.nih.gov/project_info_description.cfm?aid=9617601) | Pamela Kohler | Kenya |
| [CombinADO: a combination intervention strategy to improve health outcomes for adolescents living with HIV](https://projectreporter.nih.gov/project_info_description.cfm?aid=9618406) | Elaine Abrams | Mozambique & South Africa |
| [The BeT intervention to reduce HIV prevention and care disparities among young transwomen in Rio De Janeiro](https://projectreporter.nih.gov/project_info_description.cfm?aid=9618068) | Erin Meek | Brazil |
| [ITEST: Innovative Tools to Expand Youth-Friendly HIV Self-Testing](https://projectreporter.nih.gov/project_info_description.cfm?aid=9618360) | Juliet Iwelunmor | Nigeria |
| [Multilevel Comprehensive HIV Prevention for South African Adolescent Girls and Young Women](https://projectreporter.nih.gov/project_info_description.cfm?aid=9617086) (IMARA) | Geri Donenberg | South Africa |
| [Developing and Testing a Multi-level Package of Interventions for an Integrated Care Delivery Model of HIV Prevention and Treatment Targeting Adolescent Girls in Zambia](https://projectreporter.nih.gov/project_info_description.cfm?aid=9617501) (SHIELD & IWC) | Sujha Subramanian | Zambia |
| [Intensive Combination Approach to Rollback the Epidemic (iCARE) in Nigerian Adolescents](https://projectreporter.nih.gov/project_info_description.cfm?aid=9617899) | Babafemi Taiwo | Nigeria |
